# Adjuvant therapy provides no additional recurrence-free benefit for esophageal squamous cell carcinoma patients after neoadjuvant chemoimmunotherapy and surgery: a multi-center propensity score match study

**DOI:** 10.3389/fimmu.2024.1332492

**Published:** 2024-02-05

**Authors:** Shu-Han Xie, Li-Tao Yang, Hai Zhang, Zi-Lu Tang, Zhi-Wei Lin, Yi Chen, Zhi-Nuan Hong, Rong-Yu Xu, Wan-Li Lin, Ming-Qiang Kang

**Affiliations:** ^1^ Department of Thoracic Surgery, Fujian Medical University Union Hospital, Fuzhou, Fujian, China; ^2^ The Graduate School of Fujian Medical University, Fuzhou, Fujian, China; ^3^ Department of Thoracic Surgery, Baoji Traditional Chinese Medicine Hospital, Baoji, Shaanxi, China; ^4^ Department of Thoracic Surgery, Gaozhou People’s Hospital, Gaozhou, Guangdong, China; ^5^ Department of Thoracic Surgery, Quanzhou First Hospital, Quanzhou, Fujian, China; ^6^ Department of Thoracic Surgery, Quanzhou First Hospital Affiliated to Fujian Medical University, Quanzhou, Fujian, China; ^7^ Key Laboratory of Cardio-Thoracic Surgery (Fujian Medical University), Fujian Province University, Fuzhou, Fujian, China; ^8^ Key Laboratory of Gastrointestinal Cancer (Fujian Medical University), Ministry of Education, Fuzhou, Fujian, China; ^9^ Fujian Key Laboratory of Tumor Microbiology, Fujian Medical University, Fuzhou, Fujian, China

**Keywords:** adjuvant therapy, neoadjuvant chemoimmunotherapy, esophageal cancer, propensity score matching, recurrence-free survival

## Abstract

**Purpose:**

The need for adjuvant therapy (AT) following neoadjuvant chemoimmunotherapy (nICT) and surgery in esophageal squamous cell cancer (ESCC) remains uncertain. This study aims to investigate whether AT offers additional benefits in terms of recurrence-free survival (RFS) for ESCC patients after nICT and surgery.

**Methods:**

Retrospective analysis was conducted between January 2019 and December 2022 from three centers. Eligible patients were divided into two groups: the AT group and the non-AT group. Survival analyses comparing different modalities of AT (including adjuvant chemotherapy and adjuvant chemoimmunotherapy) with non-AT were performed. The primary endpoint was RFS. Propensity score matching(PSM) was used to mitigate inter-group patient heterogeneity. Kaplan-Meier survival curves and Cox regression analysis were employed for recurrence-free survival analysis.

**Results:**

A total of 155 nICT patients were included, with 26 patients experiencing recurrence. According to Cox analysis, receipt of adjuvant therapy emerged as an independent risk factor(HR:2.621, 95%CI:[1.089,6.310], P=0.032), and there was statistically significant difference in the Kaplan-Meier survival curves between non-AT and receipt of AT in matched pairs (p=0.026). Stratified analysis revealed AT bring no survival benefit to patients with pathological complete response(p= 0.149) and residual tumor cell(p=0.062). Subgroup analysis showed no significant difference in recurrence-free survival between non-AT and adjuvant chemoimmunotherapy patients(P=0.108). However, patients receiving adjuvant chemotherapy exhibited poorer recurrence survival compared to non-AT patients (p= 0.016).

**Conclusion:**

In terms of recurrence-free survival for ESCC patients after nICT and surgery, the necessity of adjuvant therapy especially the adjuvant chemotherapy, can be mitigated.

## 1 Introduction

Esophageal cancer accounts for approximately 50% of cancer cases in China, with over 90% diagnosed as esophageal squamous cell carcinoma (ESCC) (1, 2). Esophagectomy plays a pivotal role in the treatment of locally advanced esophageal squamous cell carcinoma (3). However, surgery alone often results in substantial recurrence and metastasis, with rates ranging from 43.3% to 50.0% (4).

Currently, the standard treatment for locally advanced ESCC involves minimally invasive esophagectomy following neoadjuvant therapy (5). However, the standard neoadjuvant therapy for locally advanced ESCC remains uncertain. Neoadjuvant chemoradiotherapy (nCRT) is commonly used in Western countries, while neoadjuvant chemotherapy (nCT) is extensively used in China and Japan (6) (7). Despite availability of these treatments, the survival of ESCC patients following neoadjuvant therapy is poor due to high recurrence rates and limited long-term survival. The 10-year results from the CROSS trial show a 63.6% disease-free survival rate in the nCRT group, with a 24.3% distant metastasis rate (8). Therefore, there is an urgent need for more effective systemic therapies to improve long-term survival outcomes. Previous study indicates enhanced prognosis in patients receiving nCRT following the addition of adjuvant chemotherapy(aCT) (9). Additionally, neoadjuvant chemoimmunotherapy (nICT) has emerged as a promising and innovative approach for locally advanced ESCC in recent years. Our center has conducted a single-arm phase II clinical trial to evaluate the safety and efficacy of nICT in the treatment of locally advanced ESCC (LA-ESCC) (10). Furthermore, the combination of pembrolizumab (a PD-1 inhibitor) with chemotherapy has been recommended as a first-line treatment for advanced EC (11). The NICE phase-II study demonstrated a 78.1% 2-year recurrence-free survival rate and a 67.9% overall survival rate after nICT (12). The CheckMate577 study revealed that adjuvant immunotherapy following nCRT and esophagectomy significantly extended median disease-free survival to 11.0 months, highlighting the therapeutic advantage of immunotherapy as a systemic treatment option (13).

However, it is imperative to elucidate whether adjuvant therapy, including aCT and adjuvant chemoimmunotherapy(aICT), is indispensable following nICT. Considering the long-term immune memory effect of immunotherapeutic agents (14, 15), we propose that postoperative adjuvant treatment might not be necessary for improved recurrence-free survival in esophageal cancer patients undergoing nICT.

## 2 Methods

### 2.1 Patient selection

This study retrospectively enrolled patients who underwent esophagectomy at three centers(Fujian Medical University Union Hospital, Quanzhou First Hospital and Gaozhou People’s Hospital) between January 1, 2019, and December 30, 2022. The inclusion criteria of this study were as follows: 1. Patients diagnosed with cT_3-4a_N_any_M_0_ or cT_1-2_N_+_M_0_ ESCC; 2. receiving at least one cycle of nICT without restrictions on the chemotherapy regimen and type of immunodrug; 3. undergoing radical resection(R0 resection); and 4. provided complete clinical and pathological information. The exclusion criteria were as follows: 1. Patients diagnosed with esophageal adenocarcinoma or other pathological type; 2. patients who underwent only exploratory surgery or jejunostomy; and 3. patients who received radiotherapy before or after surgery. The patient selection procedure is summarized in the flowchart (**Figure S1**).

### 2.2 Treatment protocols

Diagnostic and clinical staging procedures included gastroscopy, contrast-enhanced computed tomography of the neck, chest, and upper abdomen, as well as neck ultrasound. Positron emission computed tomography was performed when necessary.

The chemotherapy regimen primarily consisted of platinum in combination with paclitaxel or docetaxel, administered every three weeks. Common neoadjuvant chemotherapy regimens involved cisplatin(60 mg/m^2^) on day 1, followed by nab-paclitaxel(125 mg/m^2^) on days 1 and 8, or docetaxel(75 mg/m^2^) with cisplatin(60 mg/m^2^) on day 1. Following neoadjuvant chemotherapy, PD-1 monoclonal antibodies were administered, including camrelizumab, pembrolizumab, sintilimab, tislelizumab, or toripalimab, as detailed in our previous studies (16, 17). Generally, PD-1 inhibitors were administered every three weeks, including sintilimab at a dosage of 200 mg, toripalimab at a dosage of 240 mg, pembrolizumab at a dosage of 200 mg, tislelizuma at a dosage of 200 mg and camrelizumab at a dosage of 200 mg.

Suitable candidates for curative esophagectomy, without contraindications, typically underwent the procedure 4-8 weeks after the last dose of neoadjuvant therapy. Esophagectomy with standard 2-field or 3-field lymphadenectomy and gastric reconstruction was performed. Neck lymphadenectomy was conducted if preoperative imaging indicated suspected neck lymph node enlargement.

Postoperative adjuvant therapy was not mandatory and was applied depending on a comprehensive assessment of pathological outcomes, treatment preferences, physical condition, and physician evaluation. Adjuvant therapy regimens in this study included chemotherapy(aCT), immunotherapy(aIT), or a combination of both(aICT).

Dosages and cycles were determined by expert oncologists and thoracic surgeons, and adjusted as needed for drug-related toxicities, patient tolerance, or tumor response to treatment.

### 2.3 Follow-up and outcomes measure

In accordance with the NCCN and CSCO guideline, ESCC patients were subjected to regular follow-up examinations every 3 to 6 months within the initial two-year period. Subsequently, follow-ups were conducted at 6-month intervals from the third to fifth year, and annually thereafter. Commonly, the follow-up methods included outpatient visits and telephone interviews. Computed tomography (CT) scans is widely used as a routine examination method to monitor for recurrence of the disease during the follow-up period. If deemed necessary and possible, a PET-CT scan or biopsy will be conducted. Follow-up times were defined from the date of surgery to recurrence or the last date of follow-up. The cut-off date of the last follow-up was October 28, 2023. The follow-up endpoint in this study is recurrence-free survival, defined as the duration from surgical resection to the occurrence of local recurrence or distal metastasis. Moreover, we investigated recurrence patterns among patients after nICT. Locoregional recurrences were defined as cancer reappearance within the esophagus, at the surgical anastomosis site, or in adjacent regional lymph nodes. Distant recurrences were defined as cancer recurrence in distant organs or beyond the operative field.

### 2.4 Relevant definitions

**Figure 1:**
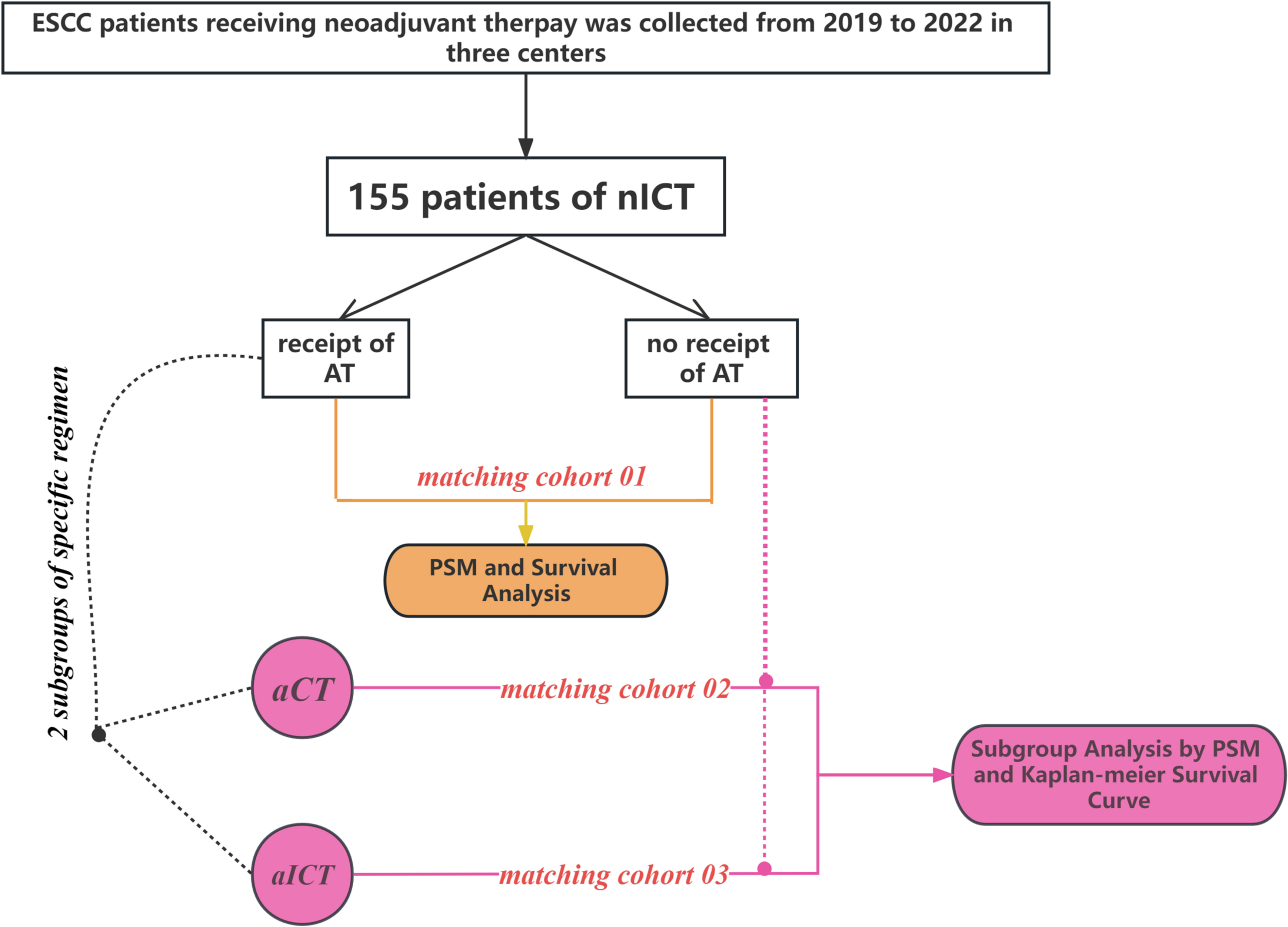
The flowchart illustrates the statistical analysis process in this study.

In this study, Propensity score matching (PSM) was performed to assess the impact of adjuvant therapy and its specific regimens on survival outcomes in distinct groups of ESCC patients. For matching cohort 1, ESCC patients with AT was compared with patients without AT. Subsequent analyses, represented by matching cohorts 2 and 3, investigated the survival advantage of specific adjuvant therapy modes compared to the absence of any adjuvant therapy. Notably, patients with adjuvant chemotherapy was compared with patients without any adjuvant therapy in matching cohort 2. Similarly, patients with adjuvant chemoimmunotherapy was compared with patients without any adjuvant therapy in matching cohort 3. Statistical analysis flow is depicted in **Figure 1**.

Additionally, The 11-month landmark method was implemented to re-evaluate the role of AT in the nICT group by excluding patients without positive outcome events and a follow-up period of no exceeding 11 months post-surgery (18–20).

### 2.5 Statistical analyses

Categorical data were presented as counts and percentages, compared using Chi-square or Fisher’s exact tests. PSM reduced bias from confounders, generating scores via logistic regression and nearest neighbor matching without replacement (caliper: 0.05). Matching parameters included pCR, ypT, ypN statuses. Matching cohorts 1 and 3 had a 1:1 ratio, cohort 2 a 1:2 ratio. Survival differences were analyzed using Kaplan-Meier curves and log-rank tests. In addition, Cox regression was performed to evaluate risk factors (variables with p< 0.05 in univariate analysis were included in the multivariate analysis, using LR stepwise regression method). The reverse Kaplan-Meier method was used to calculate the median follow-up duration. In this study, the statistical test values were calculated using the chi-square test. Data were analyzed using SPSS (v25) and R (v4.3.1). Statistical significance was set at P < 0.05.

## 3 Results

### 3.1 Baseline characteristic of ESCC patients in the nICT group

Our study included a total of 155 patients from three centers. The study consisted of 125 males (80.6%) and 30 females (19.4%). Among them, 77 patients(49.6%) received AT. All patients took a TP or DP for their neoadjuvant chemotherapy regimen. Within the AT recipients, only 20 patients receive adjuvant chemotherapy and 10 patients received adjuvant immunotherapy (aIT), while 47 patients take aICT as their adjuvant therapy regimen. The median follow-up duration of this study was 23 months (95%CI: 20.95-25.05; range:2-48 months). Detailed information about patients in nICT group is presented in **Table 1**.

**Table 1:** Clinicopathological Characteristics of patients in nICT group.

Clinicopathological characteristic	N (%)
Sex	
male	125 (80.6%)
female	30 (19.4%)
Age	
≤65	122 (78.7%)
>65	33 (21.3%)
BMI	
<18.5	18 (11.6%)
18.5-23.9	106 (68.4%)
≥24	31 (20.0%)
Smoking history	
no	66 (42.6%)
yes	89 (57.4%)
Tumor location	
upper	14 (9.0%)
middle	90 (58.1%)
lower	51 (32.9%)
Regimen of nCT	
TP/DP	155 (100.0%)
Others	0 (0%)
Type of immunodrug	
sintilimab	34 (21.9%)
toripalimab	14 (9.0%)
pembrolizumab	30 (19.4%)
tislelizumab	11 (7.1%)
camrelizumab	66 (42.6%)
Cycle of neoadjuvant therapy	
≤2	121 (78.1%)
>2	34 (21.9%)
Adjuvant therapy	
no	78 (50.3%)
yes	77 (49.7%)
Type of adjuvant therapy	
aCT	20 (26.0%)
aICT	47 (61.0%)
aIT	10 (13.0%)
Regimen of chemotherapy in aCT and aICT	
TP/DP	65 (97.0%)
Others*	2 (3.0%)
Stage	
I	67 (43.2%)
II	24 (15.5%)
IIIa	22 (14.2%)
IIIb	38 (24.5%)
IVa	4 (2.6%)
ypT stage	
T_0_	47 (30.3%)
T_1_	31 (20.0%)
T_2_	22 (14.2%)
T_3_	55 (35.5%)
ypN stage	
N_0_	91 (58.7%)
N_1_	38 (24.5%)
N_2_	22 (14.2%)
N_3_	4 (2.6%)
Pathological response	
non-pCR	119 (76.8%)
pCR	36 (23.2%)

nCT, neoadjuvant chemotherapy; aICT, adjuvant chemoimmunotherapy; aCT, adjuvant chemotherapy; aIT, adjuvant immunotherapy; pCR, pathological complete response. TP/DP: paclitaxel combined with platinum-based chemotherapy or docetaxel combined with platinum-based chemotherapy.

*2 patients take platinum +5-FU as adjuvant chemotherapy regimen.

### 3.2 Survival comparison between AT recipients and non-AT patients in the nICT group before and after PSM

**Figure 2:**
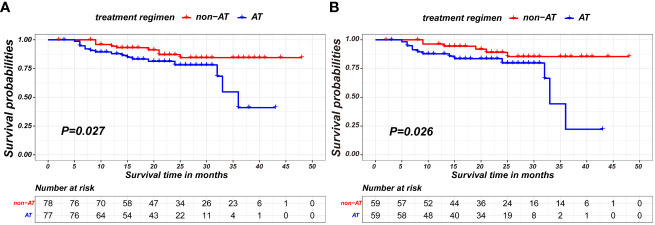
Kaplan-Meier survival curves of recurrence-free survival between non-AT and AT recipients in the nICT group before PSM **(A)** and after PSM **(B)**.

A comparison of baseline characteristics between the AT and non-AT patient populations is detailed in **Table 2**. Before PSM, AT recipients had a worse RFS compared to patients without AT (p=0.027). Similarly, Kaplan-Meier curve analysis and log-rank tests indicated statistically significant differences between patients who received AT and those who did not after PSM (p=0.026), as shown in **Figure 2**.

**Table 2:** Characteristics comparison of AT and non-AT patients in nICT group before and after matching.

	Before PSM		Statistics value	P value	After PSM		Statistics value	P value
	non-AT	AT			non-AT	AT		
**Sex**			5.762	0.016			3.576	0.059
male	57 (73.1%)	68 (88.3%)			44 (74.6%)	52 (88.1%)		
female	21 (26.9%)	9 (11.7%)			15 (25.4%)	7 (11.9%)		
**Age**			1.774	0.183			0.837	0.360
≤65	58 (74.4%)	64 (83.1%)			45 (76.3%)	49 (83.1%)		
>65	20 (25.6%)	13 (16.9%)			14 (23.7%)	10 (16.9%)		
**BMI**			0.952	0.621			1.943	0.379
<18.5	11 (14.1%)	7 (9.1%)			9 (15.3%)	5 (8.5%)		
18.5-23.9	52 (66.7%)	54 (70.1%)			42 (71.2%)	42 (71.2%)		
≥24	15 (19.2%)	16 (20.8%)			8 (13.6%)	12 (20.3%)		
**Smoking history**			1.514	0.219			0.555	0.456
no	37 (47.4%)	29 (37.7%)			27 (45.8%)	23 (39.0%)		
yes	41 (52.6%)	48 (62.3%)			32 (54.2%)	36 (61.0%)		
**Tumor location**			5.704	0.058			1.950	0.377
upper	4 (5.1%)	10 (13.0%)			4 (6.8%)	7 (11.9%)		
middle	52 (66.7%)	38 (49.4%)			37 (62.7%)	30 (50.8%)		
lower	22 (28.2%)	29 (37.7%)			18 (30.5%)	22 (37.3%)		
**Cycle of nICT**			0.539	0.463			0.457	0.499
≤2	59 (75.6%)	62 (80.5%)			45 (76.3%)	48 (81.4%)		
>2	19 (24.4%)	15 (19.5%)			14 (23.7%)	11 (18.6%)		
**Stage**			6.325	0.175			0.000	1.000
I	39 (50.0%)	28 (36.4%)			27 (45.8%)	27 (45.8%)		
II	13 (16.7%)	11 (14.3%)			11 (18.6%)	11 (18.6%)		
IIIa	9 (11.5%)	13 (16.9%)			9 (15.3%)	9 (15.3%)		
IIIb	14 (17.9%)	24 (31.2%)			12 (20.3%)	12 (20.3%)		
IVa	3 (3.8%)	1 (1.3%)			0 (0%)	0 (0%)		
**ypT stage**			2.945	0.400			0.000	1.000
T_0_	27 (34.6%)	20 (26.0%)			17 (28.8%)	17 (28.8%)		
T_1_	14 (17.9%)	17 (22.1%)			14 (23.7%)	14 (23.7%)		
T_2_	13 (16.7%)	9 (11.7%)			8 (13.6%)	8 (13.6%)		
T_3_	24 (30.8%)	31 (40.3%)			20 (33.9%)	20 (33.9%)		
**ypN stage**			6.210	0.093			0.000	1.000
N_0_	52 (66.7%)	39 (50.6%)			38 (64.4%)	38 (64.4%)		
N_1_	14 (17.9%)	24 (31.2%)			14 (23.7%)	14 (23.7%)		
N_2_	9 (11.5%)	13 (16.9%)			7 (11.9%)	7 (11.9%)		
N_3_	3 (3.8%)	1 (1.3%)			0 (0%)	0 (0%)		
**Pathological response**			2.183	0.140			0.000	1.000
non-pCR	56 (71.8%)	63 (81.8%)			45 (76.3%)	45 (76.3%)		
pCR	22 (28.2%)	14 (18.2%)			14 (23.7%)	14 (23.7%)		

nICT, neoadjuvant chemoimmunotherapy; pCR, pathological complete response; AT, adjuvant chemotherapy.

Subsequently, Cox regression analysis was conducted in unmatched pairs to analyze the risk factors affecting the RFS of nICT patients. In univariate Cox analysis, ypN status, ypT status, smoking history and AT were identified as the significant influencing factor for RFS in nICT patients. While, AT(HR:2.621, 95%CI: [1.089,6.310], P=0.032) and ypN status were significant independent risk factor for RFS in multivariate Cox analysis, as shown in **Table 3**.

**Table 3:** Univariate and multivariate Cox analysis for recurrence-free survival in a unmatched population.

	Univariate Cox analysis			Multivariate Cox analysis		
	HR	95%CI	P value	HR	95%CI	P value
**Sex**			0.112			
male	1.000					
female	0.310	[0.073,1.313]				
**age**			0.187			
≤65	1.000					
>65	0.377	[0.089,1.604]				
**smoking history**			0.042			
no	1.000					
yes	2.469	[1.034,5.894]				
**BMI**			0.451			
<18.5	1.000					
18.5-23.9	3.325	[0.444,24.933]	0.242			
>24	3.855	[0.474,31.364]	0.207			
**Location**			0.171			
Upper	1.000					
Middle	0.527	[0.146,1.909]	0.330			
lower	1.163	[0.323,4.181]	0.817			
**Cycle of nICT**			0.399			
≤2	1.000					
>2	1.484	[0.593,3.716]				
**Receipt of AT**			0.033			0.032
No	1.000			1.000		
Yes	2.460	[1.075,5.626]		2.621	[1.089,6.310]	
**ypT stage**			0.198			
T_0_	1.000					
T_1_	2.368	[0.668,8.397]	0.182			
T_2_	1.686	[0.377,7.536]	0.494			
T_3_	3.270	[1.065,10.037]	0.038			
**ypN stage**			0.005			0.014
N_0_	1.000			1.000		
N_1_	1.448	[0.525,3.992]	0.475	1.185	[0.424,3.312]	0.746
N_2_	3.624	[1.427,9.206]	0.007	3.019	[1.173,7.771]	0.022
N_3_	9.514	[2.021,44.791]	0.004	14.087	[2.821,70.342]	0.001
**Pathological response**			0.050			
non-pCR	1.000					
pCR	0.236	[0.056,0.998]				

nICT, neoadjuvant chemoimmunotherapy; AT, adjuvant chemotherapy; pCR, pathological complete response.

**Figure 3:**
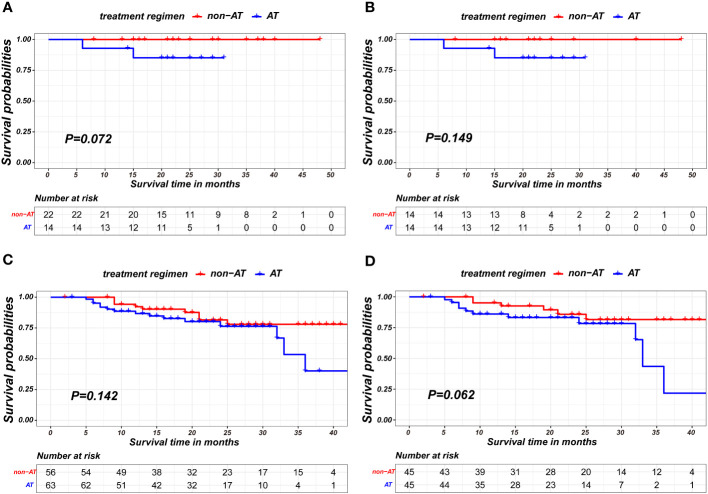
Kaplan-Meier survival curves of recurrence-free survival between non-AT and AT recipient in subgroup of pCR patients in unmatched pairs **(A)**, pCR patients in matched pairs **(B)**, non-pCR patients in unmatched pairs **(C)** and non-pCR patients in matched pairs **(D)**.

In stratified analysis, it was observed that patients with pathological complete response showed no statistically significant differences in prognosis based on the receipt of AT(p=0.072 in unmatched pairs; p= 0.149 in matched pairs), as shown in **Figures 3A, B**. Similarly, among patients with residual tumor cell(non-pCR), the receipt of AT did not result in statistically significant differences in prognosis(p=0.142 in unmatched pairs; p= 0.062 in matched pairs), as shown in **Figures 3C, D**.

### 3.3 11-month landmark analysis of the role of AT in the recipients of nICT

**Figure 4:**
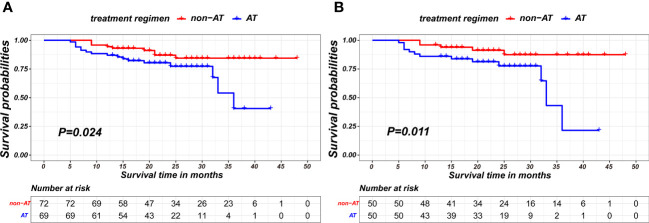
Kaplan-Meier survival curves of recurrence-free survival between non-AT and aCT patients on the 11-month landmark basis before PSM **(A)** and after PSM **(B)**.

In landmark analysis, patients without positive outcome events and a follow-up period of no more than 11 months post-surgery was excluded. The AT recipients have worse recurrence-free survival compared to patients without AT in both pre-PSM and after-PSM cohorts(P=0.024; p= 0.011, respectively), as is shown in **Figure 4**. The matching parameters in this landmark analysis including Sex, pCR, ypT and ypN status. Detailed baseline information about patients on landmark method basis is presented in **Table 4**.

**Table 4:** Characteristics comparison of AT patients and non-AT patients in the nICT group on the landmark basis before and after matching.

	Before PSM		Statistics value	P value	After PSM		Statistics value	P value
	non-AT	AT			non-AT	AT		
**Sex**			7.076	0.008			0.102	0.749
male	52 (72.2%)	62 (89.9%)			44 (88.0%)	45 (90.0%)		
female	20 (27.8%)	7 (10.1%)			6 (12.0%)	5 (10.0%)		
**Age**			1.302	0.254			0.071	0.790
≤65	55 (76.4%)	58 (84.1%)			41 (82.0%)	42 (84.0%)		
>65	17 (23.6%)	11 (15.9%)			9 (18.0%)	8 (16.0%)		
**BMI**			0.971	0.615			1.903	0.399
<18.5	10 (13.9%)	6 (8.7%)			6 (12.0%)	3 (6.0%)		
18.5-23.9	48 (66.7%)	48 (69.6%)			37 (74.0%)	36 (72.0%)		
≥24	14 (19.4%)	15 (21.7%)			7 (14.0%)	11 (22.0%)		
**Smoking history**			1.684	0.194			0.042	0.838
no	36 (50.0%)	27 (39.1%)			19 (38.0%)	20 (40.0%)		
yes	36 (50.0%)	42 (60.9%)			31 (62.0%)	30 (60.0%)		
**Tumor location**			4.453	0.108			2.346	0.309
upper	4 (5.6%)	9 (13.0%)			3 (6.0%)	7 (14.0%)		
middle	48 (66.7%)	35 (50.7%)			32 (64.0%)	26 (52.0%)		
lower	20 (27.8%)	25 (36.2%)			15 (30.0%)	17 (34.0%)		
**Cycle of nICT**			0.016	0.900			0.000	1.000
≤2	58 (80.6%)	55 (79.7%)			40 (80.0%)	40 (80.0%)		
>2	14 (19.4%)	14 (20.3%)			10 (20.0%)	10 (20.0%)		
**Stage**			4.779	0.320			1.053	1.000
I	36 (50.0%)	27 (39.1%)			24 (48.0%)	24 (48.0%)		
II	12 (16.7%)	10 (14.5%)			10 (20.0%)	9 (18.0%)		
IIIa	8 (11.1%)	9 (13.0%)			6 (12.0%)	6 (12.0%)		
IIIb	13 (18.1%)	22 (31.9%)			10 (20.0%)	10 (20.0%)		
IVa	3 (4.2%)	1 (1.4%)			0 (0%)	1 (2.0%)		
**ypT stage**			2.312	0.510			0.083	0.994
T_0_	25 (34.7%)	19 (27.5%)			16 (32.0%)	16 (32.0%)		
T_1_	13 (18.1%)	14 (20.3%)			9 (18.0%)	10 (20.0%)		
T_2_	12 (16.7%)	8 (11.6%)			8 (16.0%)	8 (16.0%)		
T_3_	22 (30.6%)	28 (40.6%)			17 (34.0%)	16 (32.0%)		
**ypN stage**			4.371	0.239			1.015	1.000
N_0_	48 (66.7%)	37 (53.6%)			34 (68.0%)	33 (66.0%)		
N_1_	12 (16.7%)	19 (27.5%)			10 (20.0%)	10 (20.0%)		
N_2_	9 (12.5%)	12 (17.4%)			6 (12.0%)	6 (12.0%)		
N_3_	3 (4.2%)	1 (1.4%)			0 (0%)	1 (2.0%)		
**Pathological response**			1.488	0.223			0.000	1.000
non-pCR	51 (70.8%)	55 (79.7%)			36 (72.0%)	36 (72.0%)		
pCR	21 (29.2%)	14 (20.3%)			14 (28.0%)	14 (28.0%)		

nICT, neoadjuvant chemoimmunotherapy; AT, adjuvant chemotherapy; pCR, pathological complete response.

### 3.4 Subgroup analysis of survival comparison between patients of non-AT and two modalities of AT

**Figure 5:**
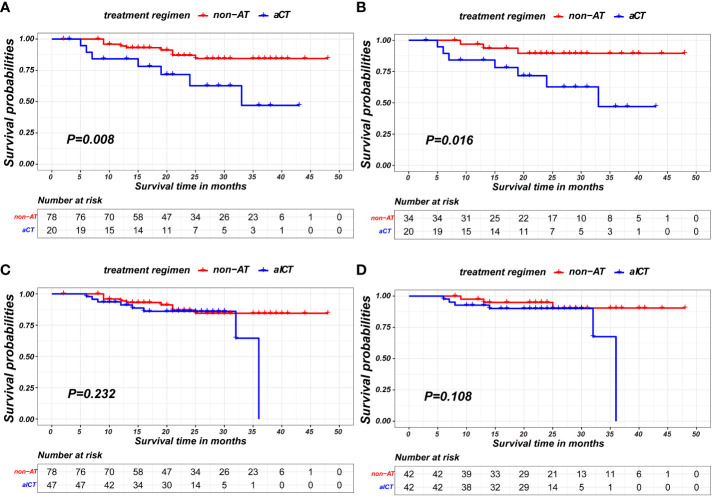
Kaplan-Meier survival curves of recurrence-free survival between patients of non-AT and two modalities of AT: **(A)** comparison of patients of non-AT with aCT before PSM; **(B)** comparison of patients of non-AT with aCT after PSM; **(C)** comparison of patients of non-AT with aICT before PSM; **(D)** comparison of patients of non-AT with aICT after PSM).

In the nICT group, both before and after PSM, patients receiving aCT exhibited poorer prognosis in terms of recurrence-free survival compared to non-AT patients (p=0.008;p= 0.016, respectively), as shown in **Figures 5A, B**. Detailed baseline information for these two matched pairs before and after matching is presented in **Table 5**.

**Table 5:** Characteristics comparison of aCT patients and non-AT patients before and after matching.

	Before PSM		Statistics value	P value	After PSM		Statistics value	P value
	non-AT	aCT			non-AT	aCT		
**Sex**			4.395	0.038			3.847	0.072
male	57 (73.1%)	19 (95.0%)			25 (73.5%)	19 (95.0%)		
female	21 (26.9%)	1 (5.0%)			9 (26.5%)	1 (5.0%)		
**Age**			1.004	0.389			0.064	1.000
≤65	58 (74.4%)	17 (85.0%)			28 (82.4%)	17 (85.0%)		
>65	20 (25.6%)	3 (15.0%)			6 (17.6%)	3 (15.0%)		
**BMI**			1.244	0.681			5.111	0.101
<18.5	11 (14.1%)	1 (5.0%)			5 (14.7%)	1 (5.0%)		
18.5-23.9	52 (66.7%)	15 (75.0%)			28 (82.4%)	15 (75.0%)		
≥24	15 (19.2%)	4 (20.0%)			1 (2.9%)	4 (20.0%)		
**Smoking history**			1.965	0.161			1.518	0.218
no	37 (47.4%)	6 (30.0%)			16 (47.1%)	6 (30.0%)		
yes	41 (52.6%)	14 (70.0%)			18 (52.9%)	14 (70.0%)		
**Tumor location**			1.857	0.523			1.888	0.543
upper	4 (5.1%)	0 (0%)			3 (8.8%)	0 (0%)		
middle	52 (66.7%)	12 (60.0%)			18 (52.9%)	12 (60.0%)		
lower	22 (28.2%)	8 (40.0%)			13 (38.2%)	8 (40.0%)		
**Cycle of nICT**			0.267	0.606			0.078	0.780
≤2	59 (75.6%)	14 (70.0%)			25 (73.5%)	14 (70.0%)		
>2	19 (24.4%)	6 (30.0%)			9 (26.5%)	6 (30.0%)		
**Stage**			7.180	0.158			0.499	0.931
I	39 (50.0%)	6 (30.0%)			12 (35.3%)	6 (30.0%)		
II	13 (16.7%)	3 (15.0%)			6 (17.6%)	3 (15.0%)		
IIIa	9 (11.5%)	2 (10.0%)			4 (11.8%)	2 (10.0%)		
IIIb	14 (17.9%)	9 (45.0%)			12 (35.3%)	9 (45.0%)		
IVa	3 (3.8%)	0 (0%)			0 (0%)	0 (0%)		
**ypT stage**			4.697	0.217			0.193	1.000
T_0_	27 (34.6%)	4 (20.0%)			8 (23.5%)	4 (20.0%)		
T_1_	14 (17.9%)	5 (25.0%)			9 (26.5%)	5 (25.0%)		
T_2_	13 (16.7%)	1 (5.0%)			2 (5.9%)	1 (5.0%)		
T_3_	24 (30.8%)	10 (50.0%)			15 (44.1%)	10 (50.0%)		
**ypN stage**			4.989	0.196			0.470	0.872
N_0_	52 (66.7%)	9 (45.0%)			18 (52.9%)	9 (45.0%)		
N_1_	14 (17.9%)	7 (35.0%)			9 (26.5%)	7 (35.0%)		
N_2_	9 (11.5%)	4 (20.0%)			7 (20.6%)	4 (20.0%)		
N_3_	3 (3.8%)	0 (0%)			0 (0%)	0 (0%)		
**Pathological response**			1.461	0.227			0.064	1.000
non-pCR	56 (71.8%)	17 (85.0%)			28 (82.4%)	17 (85.0%)		
pCR	22 (28.2%)	3 (15.0%)			6 (17.6%)	3 (15.0%)		

nICT, neoadjuvant chemoimmunotherapy; aCT, adjuvant chemotherapy; AT, adjuvant chemotherapy; pCR, pathological complete response.

Conversely, no statistically significant differences were observed between non-AT patients and those receiving aICT before and after PSM (p=0.232; p= 0.108, respectively), as illustrated in **Figures 5C, D**. The baseline information for non-AT and aICT patients before and after matching is presented in **Table 6**.

**Table 6:** Characteristics comparison of aICT patients and non-AT patients before and after matching.

	Before PSM		Statistics value	P value	After PSM		Statistics value	P value
	non-AT	aICT			non-AT	aICT		
**Sex**			2.442	0.118			1.235	0.266
male	57 (73.1%)	40 (85.1%)			32 (76.2%)	36 (85.7%)		
female	21 (26.9%)	7 (14.9%)			10 (23.8%)	6 (14.3%)		
**Age**			1.254	0.263			0.283	0.595
≤65	58 (74.4%)	39 (83.0%)			32 (76.2%)	34 (81.0%)		
>65	20 (25.6%)	8 (17.0%)			10 (23.8%)	8 (19.0%)		
**BMI**			0.902	0.637			2.349	0.329
<18.5	11 (14.1%)	4 (8.5%)			6 (14.3%)	3 (7.1%)		
18.5-23.9	52 (66.7%)	34 (72.3%)			32 (76.2%)	31 (73.8%)		
≥24	15 (19.2%)	9 (19.1%)			4 (9.5%)	8 (19.0%)		
**Smoking history**			0.090	0.765			0.048	0.827
no	37 (47.4%)	21 (44.7%)			20 (47.6%)	19 (45.2%)		
yes	41 (52.6%)	26 (55.3%)			22 (52.4%)	23 (54.8%)		
**Tumor location**			6.187	0.045			1.191	0.601
upper	4 (5.1%)	8 (17.0%)			3 (7.1%)	6 (14.3%)		
middle	52 (66.7%)	23 (48.9%)			25 (59.5%)	22 (52.4%)		
lower	22 (28.2%)	16 (34.0%)			14 (33.3%)	14 (33.3%)		
**Cycle of nICT**			1.595	0.207			1.844	0.175
≤2	59 (75.6%)	40 (85.1%)			31 (73.8%)	36 (85.7%)		
>2	19 (24.4%)	7 (14.9%)			11 (26.2%)	6 (14.3%)		
**Stage**			6.142	0.216			0.000	1.000
I	39 (50.0%)	19 (40.4%)			19 (45.2%)	19 (45.2%)		
II	13 (16.7%)	5 (10.6%)			5 (11.9%)	5 (11.9%)		
IIIa	9 (11.5%)	9 (19.1%)			7 (16.7%)	7 (16.7%)		
IIIb	14 (17.9%)	14 (29.8%)			11 (26.2%)	11 (26.2%)		
IVa	3 (3.8%)	0 (0%)			0 (0%)	0 (0%)		
**ypT stage**			0.641	0.887			0.000	1.000
T_0_	27 (34.6%)	15 (31.9%)			13 (31.0%)	13 (31.0%)		
T_1_	14 (17.9%)	9 (19.1%)			9 (21.4%)	9 (21.4%)		
T_2_	13 (16.7%)	6 (12.8%)			6 (14.3%)	6 (14.3%)		
T_3_	24 (30.8%)	17 (36.2%)			14 (33.3%)	14 (33.3%)		
**ypN stage**			5.997	0.113			0.000	1.000
N_0_	52 (66.7%)	24 (51.1%)			24 (57.1%)	24 (57.1%)		
N_1_	14 (17.9%)	14 (29.8%)			12 (28.6%)	12 (28.6%)		
N_2_	9 (11.5%)	9 (19.1%)			6 (14.3%)	6 (14.3%)		
N_3_	3 (3.8%)	0 (0%)			0 (0%)	0 (0%)		
**Pathological response**			0.739	0.390			0.000	1.000
non-pCR	56 (71.8%)	37 (78.7%)			32 (76.2%)	32 (76.2%)		
pCR	22 (28.2%)	10 (21.3%)			10 (23.8%)	10 (23.8%)		

nICT, neoadjuvant chemoimmunotherapy; aICT, adjuvant chemoimmunotherapy; AT, adjuvant chemotherapy; pCR, pathological complete response.

### 3.5 Recurrence patterns

**Figure 6:**
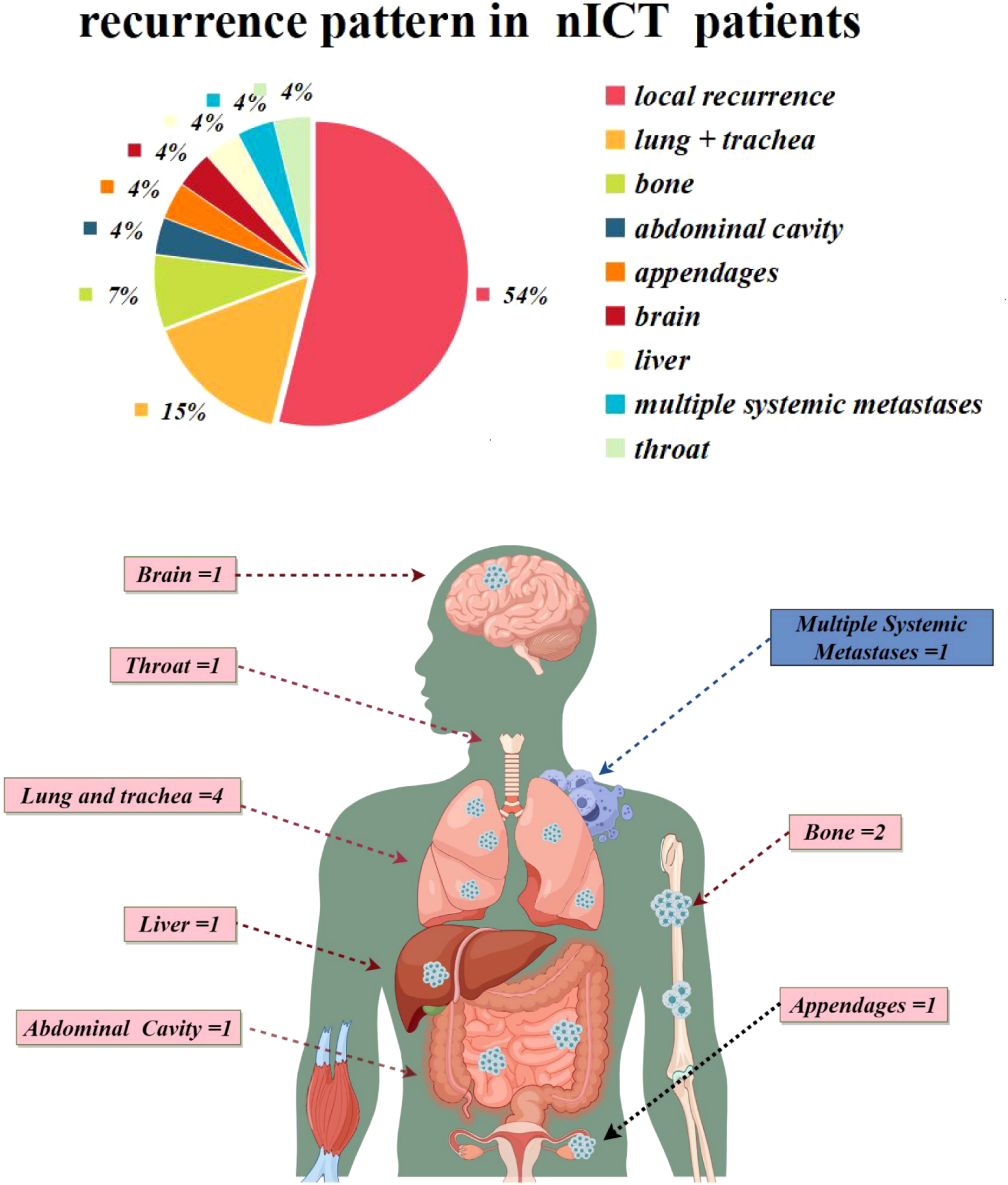
Recurrence patterns in the nICT group.

Within the nICT group, 26 patients experienced recurrence. The median time to recurrence was 12.5 months. Specifically, 14 patients had locoregional recurrences, 11 patients had distant metastasis, and 1 patients experienced both locoregional recurrence and distant metastasis. Additionally, 2 patients developed supraclavicular lymph node metastasis, classified as a locoregional recurrence in our study, as shown in **Figure 6**.

## 4 Discussion

In recent years, immunotherapy has increasingly been used for esophageal cancer patients, especially those with locally advanced stages. However, the necessity and benefits of adjuvant therapy for ESCC patients after nICT and surgery remain contentious in international medical consensus. Given the reported finding that nICT does not increase postoperative complications (21), our study is keen to investigate the prognostic factors influencing the survival of esophageal cancer patients undergoing nICT and evaluate the necessity of adjuvant therapy and different AT modalities (including aCT and aICT) in order to better guide the selection of treatment and surveillance model after nICT and surgery. In this study, data from three centers of 155 nICT cases were analyzed. After propensity score matching, there were no statistically significant differences observed in baseline characteristics between patients who received AT and those who did not. According to the Cox analysis and Kaplan-Meier curve analysis, the addition of adjuvant therapy significantly compromised the recurrence-free survival rate of ESCC following nICT in our study. These findings suggest that the administration of adjuvant therapy had a detrimental impact on recurrence-free survival following nICT and the unnecessity of AT in this patient population. This aligns with prior research indicating patients receiving adjuvant therapy exhibited a significantly diminished disease-free survival following resection and neoadjuvant chemoradiation, in comparison to those not undergoing adjuvant therapy (22).

Previous studies have reported that ESCC patients with a pathological complete response have higher 5-year overall survival than incomplete responders (23). The correlation between pathological response and prognosis influences the choice of postoperative treatment. However, the recommendation of adjuvant therapy for ESCC patients exhibiting diverse pathological responses remains a subject of ongoing controversy (9, 24). Therefore, stratified analysis was conducted in our study to evaluate survival outcomes of AT among patients achieving pCR and those with residual pathological tumor cells (ypT+ status or/and ypN+ status) after nICT. Interestingly, AT did not significantly enhance recurrence-free survival benefits for pCR cases, indicating that these individuals can adopt a “watch and see” follow-up strategy, consistent with the current postoperative follow-up strategy (25, 26). However, in cases with incomplete response, the survival advantages of AT were still not found to be statistically significant. Previous studies have demonstrated that the administration of postoperative chemotherapy does not confer any additional survival benefit to patients with lymph node metastasis following neoadjuvant chemotherapy, which aligns with our study (27). The clinical trial CheckMate-577 showed that nivolumab, as an adjuvant agent, can lead to longer disease-free survival for patients with residual pathological tumors after nCRT and surgery. However, there is insufficient evidence and research to substantiate the necessity of AT in ESCC patients who had received nICT and exhibit positive postoperative pathological findings. Previous research has demonstrated that, in comparison to as adjuvants, immune checkpoint inhibitors (ICIs) offer distinct advantages as neoadjuvant therapy for eradicating distant metastases (28). This potentially explains the improved recurrence-free survival observed in nICT cases without AT of our study, even in cases of lymph node metastasis or residual tumor cells.

Administration of different adjuvant therapy regimens may exert varying benefits in terms of recurrence-free survival of ESCC patients. Therefore, whether different adjuvant therapy regimens (aCT and aICT) could confer survival benefits to nICT cases was analyzed. In this study, the patients receiving aICT did not show any statistically significant differences compared to those without any AT. However, it was noted that recipients of aCT exhibited inferior survival rates compared to non-AT individuals following nICT and surgery in both pre-match and post-match cohorts, suggesting the absence of requirement for aCT. Previous research conducted by Yan demonstrated that postoperative adjuvant chemotherapy is not necessary for reducing recurrence in patients who have undergone neoadjuvant chemotherapy and a trend towards inferior disease-free survival was observed in patients who underwent adjuvant therapy (29), aligning with our study’s perspective on the need for adjuvant chemotherapy following neoadjuvant therapy. In addition, it is widely acknowledged that not all patients derive benefits from chemotherapy. Considering the potential impact of esophagectomy on patients, factors such as impaired postoperative food intake and swallowing ability, physiological and psychological stress, as well as postoperative complications, compromise the immune system of individuals with esophageal cancer after surgery (30–32). Consequently, the adverse effects of chemotherapy may further impede an already compromised immune system’s capacity to effectively recognize and target cancer cells, thereby diminishing the efficacy of chemotherapy or immunotherapy and leading to cancer recurrence. In addition, administration of neoadjuvant therapy may potentially suppress the responsiveness of patients towards subsequent systemic therapy post-surgery (33, 34). In this study, we identified adjuvant therapy and higher ypN status as independent risk factors of recurrence in ESCC patients following nICT and surgery. Furthermore, subgroup analysis revealed that patients receiving adjuvant chemotherapy exhibited a poorer prognosis compared to those who did not receive any adjuvant therapy. This may be due to the outweighing side effects of chemotherapy compared to its survival benefit. These findings suggest that in our clinical practice, a close follow-up strategy is preferable over continued administration of adjuvant therapy, particularly chemotherapy, for patients undergoing nICT and surgery.

For the recurrence pattern in nICT patients, the proportion of local recurrence and distant metastasis was 54% and 46%, respectively. However, the majority of patients with distant recurrence had bone metastases and respiratory system metastases, suggesting that in addition to routine CT examination, PET/CT or bone scintigraphy may help to facilitate early detection of distant metastasis.

To the best of our knowledge, this study represents the first analysis of the survival benefit associated with adjuvant therapy in ESCC patients with nICT following surgery. According to previous research, the median time to recurrence for ESCC after nCRT is approximately 11 months (35–37), which is similar with our study. Therefore, the follow-up period in this study is adequate to reflect the effectiveness of adjuvant treatment for ESCC for recurrence. Additionally, to further mitigate the potential bias caused by shorter follow-up durations compared to the median recurrence time, a sensitivity analysis(landmark method) was conducted on patients with follow-up durations exceeding 11 months. The results of this landmark analysis mirrored those of the primary analysis, suggesting that the observed lack of benefit from adjuvant therapy is robust and reliable.

Despite the implementation of rigorous inclusion and exclusion criteria, as well as propensity score matching to ensure baseline comparability, the inherent limitations of a retrospective study design may introduce some degree of bias. Additionally, the majority of patients in our study were treated with the TP/DP regimen-based protocol for neoadjuvant and adjuvants and received no more than two cycles of treatment. Consequently, conducting subgroup analysis regarding chemotherapy regimens and cycles was not feasible in this study. We are currently making efforts to collaborate with more institutions to expand our database and plan to conduct subgroup analyses in future studies. Since the incidence of ESCC is more than 90% in Asian populations, it is important to note that this study specifically focused on patients diagnosed with esophageal squamous cell carcinoma; thus, the applicability of our research findings to patients with adenocarcinoma remains uncertain.

## Conclusion

In terms of recurrence-free survival, the need for postoperative adjuvant therapy can be reduced for patients who have undergone nICT and surgery. Meanwhile, the adverse effects of postoperative adjuvant chemotherapy for patients already receiving nICT appear to outweigh its therapeutic benefits in preventing recurrence. A well-designed prospective study on a large scale is necessary to validate these findings.

## Data availability statement

The raw data supporting the conclusions of this article will be made available by the authors at reasonable request.

## Ethics statement

The studies involving humans were approved by Fujian Medical University Union Hospital (Project identification code: 2023KY241). The studies were conducted in accordance with the local legislation and institutional requirements. The ethics committee/institutional review board waived the requirement of written informed consent for participation from the participants or the participants’ legal guardians/next of kin because this is a retrospective study.

## Author contributions

SX: Conceptualization, Data curation, Formal analysis, Methodology, Software, Supervision, Writing – original draft, Writing – review & editing. LY: Conceptualization, Data curation, Formal analysis, Methodology, Writing – original draft, Writing – review & editing. HZ: Data curation, Formal analysis, Investigation, Writing – original draft. ZT: Data curation, Investigation, Writing – original draft. ZL: Data curation, Investigation, Software, Writing – original draft. YC: Data curation, Investigation, Software, Writing – original draft. ZH: Conceptualization, Data curation, Supervision, Writing – review & editing. RX: Conceptualization, Data curation, Supervision, Writing – review & editing. WL: Conceptualization, Data curation, Supervision, Writing – review & editing. MK: Conceptualization, Data curation, Supervision, Writing – review & editing.
