# Supplementary figures and images for: Adjuvant therapy provides no additional recurrence-free benefit for esophageal squamous cell carcinoma patients after neoadjuvant chemoimmunotherapy and surgery: a multi-center propensity score match study
Source: Front Immunol. 2024 Feb 5;15:1332492. doi: 10.3389/fimmu.2024.1332492 (PMC10875462; doi:10.3389/fimmu.2024.1332492)

**Supplementary material**

**
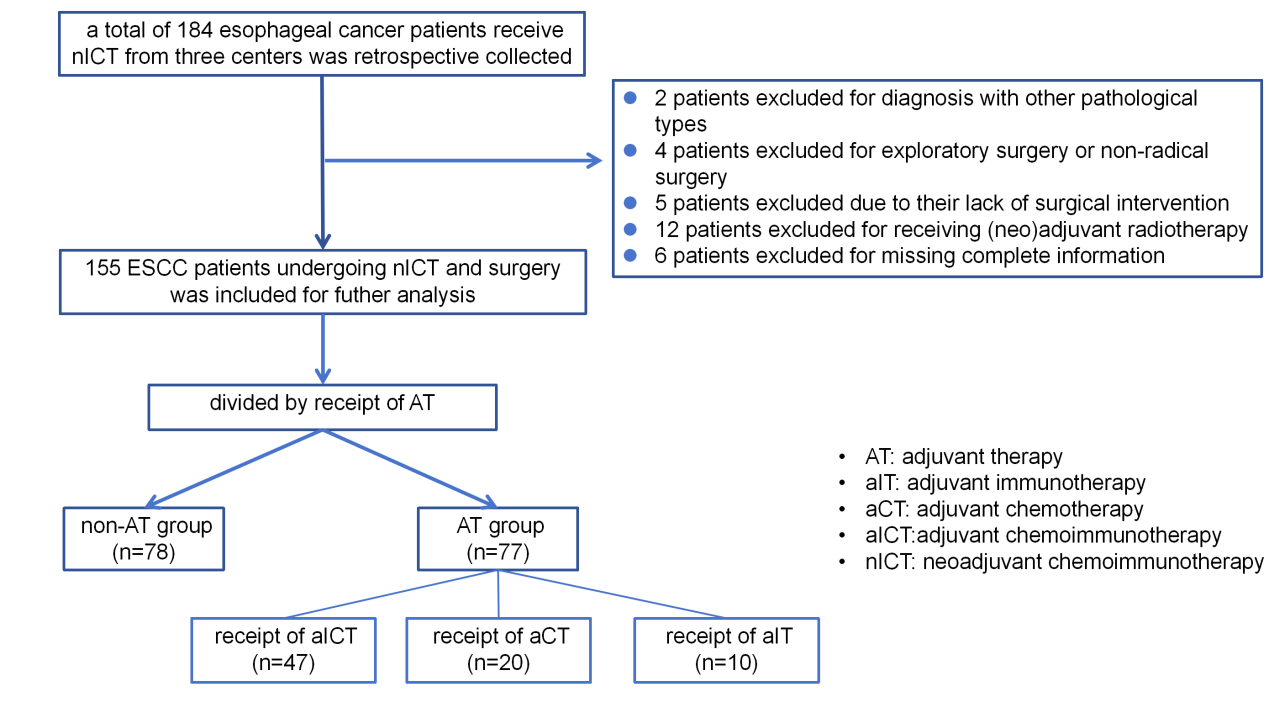
**

**Figure S1.** The flowchart of patient selection in this study.

Supplement: Supplementary file 1 [file DataSheet_1.docx]
